# The compared study about femoral stem malalignment with or without the special curved rasp during DAA total hip arthroplasty

**DOI:** 10.1186/s12891-023-06409-7

**Published:** 2023-04-22

**Authors:** Bei Lin, Yiping Lan, Zhiming Lu, Shiwei Xie, Feitai Lin, Yan Weng, Eryou Feng, Jinhua Chen

**Affiliations:** 1 Department of Arthrosis Surgery, Fuzhou Second Hospital, Fuzhou, China; 2 The Department of Clinical Medicine, Fujian Medical University, Fuzhou, China; 3 Fuzhou Second Hospital Affiliated to Xiamen University, Fuzhou, China; 4 Follow-Up Center, Fujian Medical University Union Hospital, Fuzhou, China

**Keywords:** Total hip arthroplasty, Tri-Lock (BPS) stem, Direct anterior approach, Prosthesis placement angle, Radiological analysis, WOMAC score

## Abstract

**Objective:**

To investigate whether the application of a curved rasp on the femoral side is effective in reducing the incidence of stem malalignment in total hip replacement with direct anterior approach (DAA-THA), followed by the analysis of the independent risk factors affecting stem malalignment.

**Methods:**

Retrospective analysis was carried out covering 160 patients undergoing DAA-THA from January 2019 to December 2021, with Tri-Lock (BPS, Depuy) stem applied in all 113 patients were screened according to inclusion and exclusion criteria. The data of gender, age, body mass index, preoperative diagnoses, Dorr classification, FAR ratio, pelvic morphology ratio, WOMAC scores, were analyzed to explore the independent factors influencing the malalignment of the femoral prosthesis implantation. Then data of patients were divided into group A and group B according to whether the curved rasp was taken during the operation. The chi-square test was performed to compare the incidence of femoral stem malalignment between the two groups.

**Results:**

There revealed two independent risk factors: BMI and FAR ratio that affected femoral stem malalignment. The increased BMI was associated with a higher probability of femoral stem malalignment (P<0.05), the probability of malalignment of femoral stem in FAR ratio<1 was 1.15 times higher than that in FAR>1(OR = 1.15, 95% CI: 1.03–1.28, P<0.05). Further grouping analysis showed that the incidence of femoral stem malalignment in patients with intraoperative application of curved rasp was 27%, while in patients without curved rasp, the incidence of femoral stem malalignment increased significantly to 48.7%(P<0.05). The placement angle of prosthesis in group A was significantly better than that in group B, especially mild femoral stem malalignment (0%) and severe femoral stem malalignment (2.70%), and the difference was statistically significant (P < 0.05). There found no significant difference in age, gander, intraoperative complications and last follow-up assessment of WOMAC scores between the two groups of patients.

**Conclusions:**

In DAA-THA, BMI and FAR ratio act as the independent risk factors for femoral stem malalignment. Intraoperative use of a curved rasp significantly reduces the incidence of malalignment of the femoral stem.

**Supplementary Information:**

The online version contains supplementary material available at 10.1186/s12891-023-06409-7.

## Introduction

With the increasing development of total hip arthroplasty (THA), higher-quality repair effects are constantly pursued by both doctors and patients. However, the high-quality total hip replacement not only requires the skilled surgeon, but also influenced by the patient’s own physiological anatomical configuration. In THA, femoral stem malalignment is considered critical for both survival and prognosis. Several studies have demonstrated the association of malalignment of the femoral stem with poor clinical outcomes and complications such as aseptic loosening, secondary sinking [1–3], and thigh pain [4]. Therefore, the angle of intraoperative femoral stem placement plays an imoportant role. A large amount of attempts have been performed to uncover the predictors with significant impact on this outcome, several of which affect the placement of the prosthesis, in particular the anatomical shape of the femur [5–7], the morphology of the pelvis [8], surgical approach [9], prosthesis shape and femoral preparation instruments [10, 11]. The selection and use of appropriate instrumentation for lateral femoral preparation provides significant relevance to the success of femoral stem placement and surgery. Therefore, in this study, the surgeon designed a curved rasp based on his clinical experience. The curved rasp is made of stainless steel and consists of a rasp and handle. The curved rasp has curved and granular teeth at the front end, which are designed for grinding the intramedullary cancellous bone before the large trochanter after the opening of the medullary cavity. Good preparation of the femur side facilitates the smooth implantation of the femoral stem and reduces the incidence of femoral stem malalignment.

The use of short-stem THA systems has developed rapidly over the past few years due to various theoretical advantages [12]. Referring to the literature, the emergence of short stem in recent years is resulted by relatively less surgical trauma, relatively more bone retention, and ease of revision compared to standard straight stem [13]. In contrast to the short stem, conventional long stem or stem-end fixation of the femur could result in unnecessary stress masking, enlargement of effective joint space, aseptic loosening, and potential bone loss, which may induce the failure of preserving enough intact bone for revision surgery. Therefore, the Tri-Lock (BPS, Depuy) femoral prosthesis was created. The design of Tri-Lock (BPS), not only covers the advantages of the short-stem [14], but also has a highly porous and roughened coating (Gription), which leads to mechanical integrity and long-term biological fixation [15]. Several studies have reported the good clinical outcomes and high survival rates of the Tri-lock (BPS) femoral stem, even at more than 15 years of follow-up [16, 17]. This therapeutic advantage of the Tri-lock (BPS) femoral stem reaches the agreement with the current idea of consistent, precise, and individualized treatment, serving as a practical and effective option for clinical practice.

DAA-THA is gaining increasing popularity among European and American joint surgeons. Compared to traditional surgical approaches, this approach is a intermediate approach indeed between the nerve interface and the muscle gap [18, 19]. This technique could achieve the protection of the hip abductors, posterior capsule, and short external rotators, with the advantages of less soft tissue injury, minimal bleeding, no contraindicated postoperative positions, and rapid recovery [20], contributing to increasing popularity among patients and surgeons in Asian countries [21]. Despite the growing universal of DAA, high rates of complications have also been reported in certain series, involving intraoperative femoral fractures, lateral femoral cutaneous nerve dysfunction, wound issues and prosthetic joint infections, especially early in the learning curve [22]. The most challenging aspect lies the lateral femoral exposure and preparation, and the short stem could contribute in this phase to reducing the incidence of complications associated with femoral preparation [23]. Therefore, this study covered two objectives: (1) to explore the risk factors affecting femoral stem malalignment in preparation for preoperative planning, and (2) to investigate whether the application of a curved rasp in THA for DAA is effective in reducing the rate of femoral stem placement in malalignment.

## Patients and methods

### Ethics approval and consent to participate

This study obtained the approval from the Ethics Committee of Fuzhou Second Hospital affiliated to Xiamen University (Approval number:2,022,008), and was performed in accordance with the ethical standards of the Declaration of Helsinki of 1964. Informed consent was obtained in written form from all eligible patients.

### Inclusion and exclusion criteria

The inclusion criteria were: (i) DAA-THA conducted at our hospital without significant contraindications to surgery; (ii) Tri-lock (BPS) stem used in THA; (iii) those could providing complete basic data and imaging data; (iv) no previous femoral upper end bone defect, no femoral tumor, history of tuberculosis, etc.; (v) no other hip surgery in the past; and (vi) those with an ideal degree of cooperation.

The exclusion criteria were: (i) pathological fractures; (ii) those with severe underlying diseases and mental illnesses; (iii) Dorr classification type C; (iv) bilateral total hip replacement in one-stage; (vi) ambiguous base data or imaging data; (vii) lost in follow-up.

### Patients

The first batch of 113 unilateral patients with contralateral normal hip by DAA-THA with Tri-lock (BPS) stem from January 2019 to December 2021 at the Joint Surgery Center of Fuzhou Second Hospital in China were enrolled in the retrospective study, where over 160 cases of DAA-THA were performed by the submission date. Of these patients, 32 were lost to follow-up, 7 were Dorr type C and 8 were excluded because of ambiguous underlying data or imaging.

### Surgical technique

All procedures were performed by the same fellowship-trained joint surgeon with his medical team in a trans-direct anterior approach. This primary surgeon had passed the DAA learning curve and revealed extensive posterior-lateral hip arthroplasty and revision capabilities.

### Anesthesia and position

Each patient was positioned in a supine pose on a fracture table (Hana Table, Union City, CA) under general anesthesia and femoral nerve and sciatic nerve block anesthesia. The pubic symphysis of each patient was positioned directly at the folding mark of the table.

### Surgical instrument preparation

**Fig. 1:**
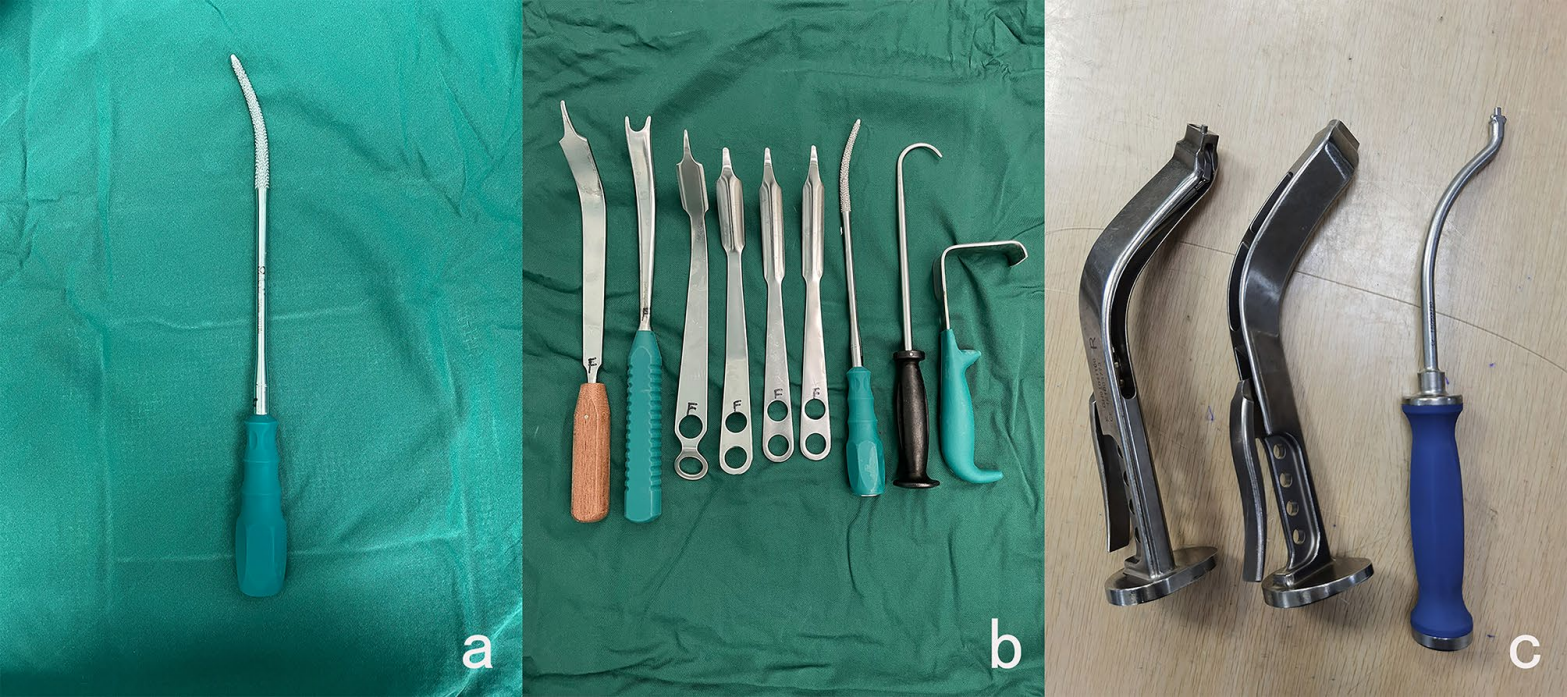
**DAA surgical instruments.** **a** is our main introduction of curved rasp. **b** is 6 Hoffman hooks, curved rasp, bone hook, skin retractor. **c** is Offset eccentric handle

Depending on years of clinical experience, a set of practical DAA 9-piece sets of anterior auxiliary retractors was designed, composed of a curved rasp, 6 Hoffman hooks, and handle with Offset eccentric (Fig. 1). The curvature of the hooks was designed according to the intraoperative position, with each Hoffman hook slightly curved upward on both sides to avoid cutting damage to soft tissues. The curved rasp can assist to remove bone anterior to the greater trochanter, effectively decreasing the incidence of femoral prosthesisvalgus-varus. The handle with Offset eccentric can contribute to alleviating the impact of the tool on the anterior superior iliac spine and local soft tissue contusion, avoiding the occurrence of femoral side complications during intraoperative manipulation.

### Surgical procedure

**Fig. 2:**
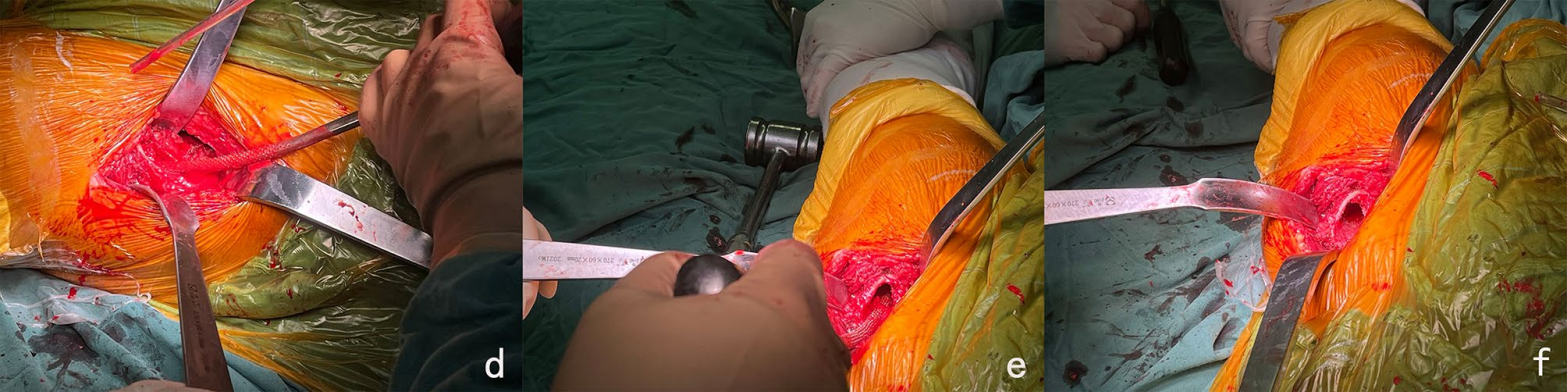
**Intraoperative management of the femoral side.** A curved rasp was taken to fully remove the intramedullary cancellous bone anterior to the greater trochanter until the lateral cortical bone of the femur at the plane of the lesser trochanter was palpated

An incision was created on the anterior side of the hip, 2 cm from the anterior superior iliac spine inferiorly and posteriorly, with the distal end pointing to the fibular head, about 8 cm in length. The superficial fascia, tensor fascia lata, and joint capsule were opened layer by layer. Femoral neck osteotomy was performed according to preoperative plan, so as to fully expose the acetabulum, the surrounding joint capsule was cleaned. The acetabulum was reamed, and the biological acetabular prosthesis was place at an appropriate anteversion angle; the tail end of the operating table was slightly lowered by about 30 degrees, the joint capsule above the proximal femur was fully released piriform fossa, with the insertion point of the obturator external muscle retained, the proximal femur with the assistance of the bone hook was lifted, with the patient maintained in extreme inward and outward rotation position. After opening the proximal femur, a curved rasp was taken to fully remove the intramedullary cancellous bone anterior to the greater trochanter until the lateral cortical bone of the femur at the plane of the lesser trochanter was palpated. The femoral canal was then prepared using an eccentric curve rasp (Fig. 2). The femoral stem prosthesis was installed, with the anteversion angle maintained parallel to the posterior cortex of the femoral neck osteotomy surface. The artificial femoral head was installed and the hip joint was reset. The incision was checked and rinsed, and the incision was sutured layer by layer.

### Postoperative reconstruction

A standard protocol covering multimodal analgesia, physiotherapy, and enhanced recovery was launched immediately following the conclusion of surgery. Patients were instructed to wear and take off socks by themselves on the first day and encouraged to walk using crutches as soon as possible based on their own conditions. Clinical follow-up was performed at 1 month, 3 months, 1 year and every year thereafter, in which X-rays were taken on postoperative day 1 and at each follow-up visit.

### Radiograph data

Anteroposterior radiographs of the pelvis in the supine position and lateral radiographs of the bilateral hip joints of each patient were taken, with 15°of bilateral hip internal rotation and no abduction.

In order to alleviate the human subjective measurement error, each radiograph was evaluated by 2 physicians and the means of the two values were obtained for further measurements. All imaging measurements were performed using the Medical Image Archive and Communication System or Mimics 20.0.

## Measurement details

### Dorr classification

According to the Dorr classification [24], we measured the patient’s preoperative X-rays and divided the femoral bone marrow cavity into two types: type A and B. Type A bone has radiologically a good structural funnel shape and narrow lateral diaphyseal canal isthmus. Type B bone shows loss from the proximal cortex of bone, as shown by the relatively higher intramedullary canal diameter and cortical index on the lateral radiograph.

### FAR ratio

**Fig. 3:**
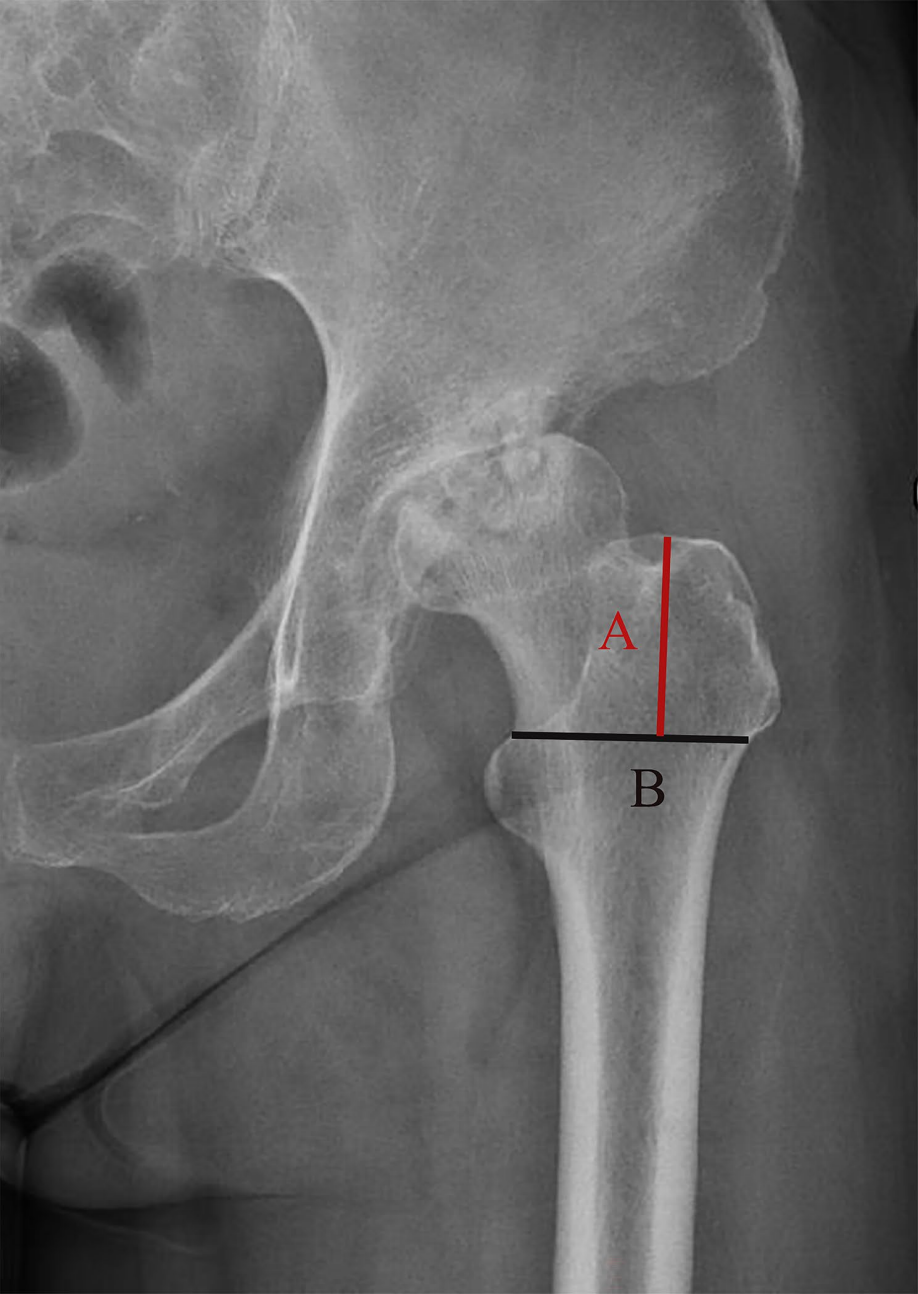
Femoral access ratio (FAR) (A/B)

The FAR ratio was measured on anterior and lateral radiographs of the hip joint respectively, which consisted of 2 measurements by two physicians on a proximal femoral flat slice. The B line was a horizontal that beginning at the proximal end of the lesser trochanter (LT) and spanning the width of the proximal femur from the external cortex to the external cortex. The A line was a line perpendicular to the B line extending from the uppermost part of the greater trochanter (GT) down to the B line. The FAR was calculated through dividing the A line by the B line (Fig. 3). This made it free from the requirement for absolute measurements and allowed easy comparison of observers at different magnifications [25]. The FAR ratio were measured according to the frontal and lateral X-ray plain films of the hip joint before operation.

### Pelvic morphometric

By preoperatively measuring the pelvic morphometric ratio, that was, the line connecting the widest part of the ilium and the most distal part of the ischium, the ratio of the two lines was greater than 3:1, indicating that the ilium wing was more likely to hinder the implantation of the stem [8].

### Prosthesis implantation angle

**Fig. 4:**
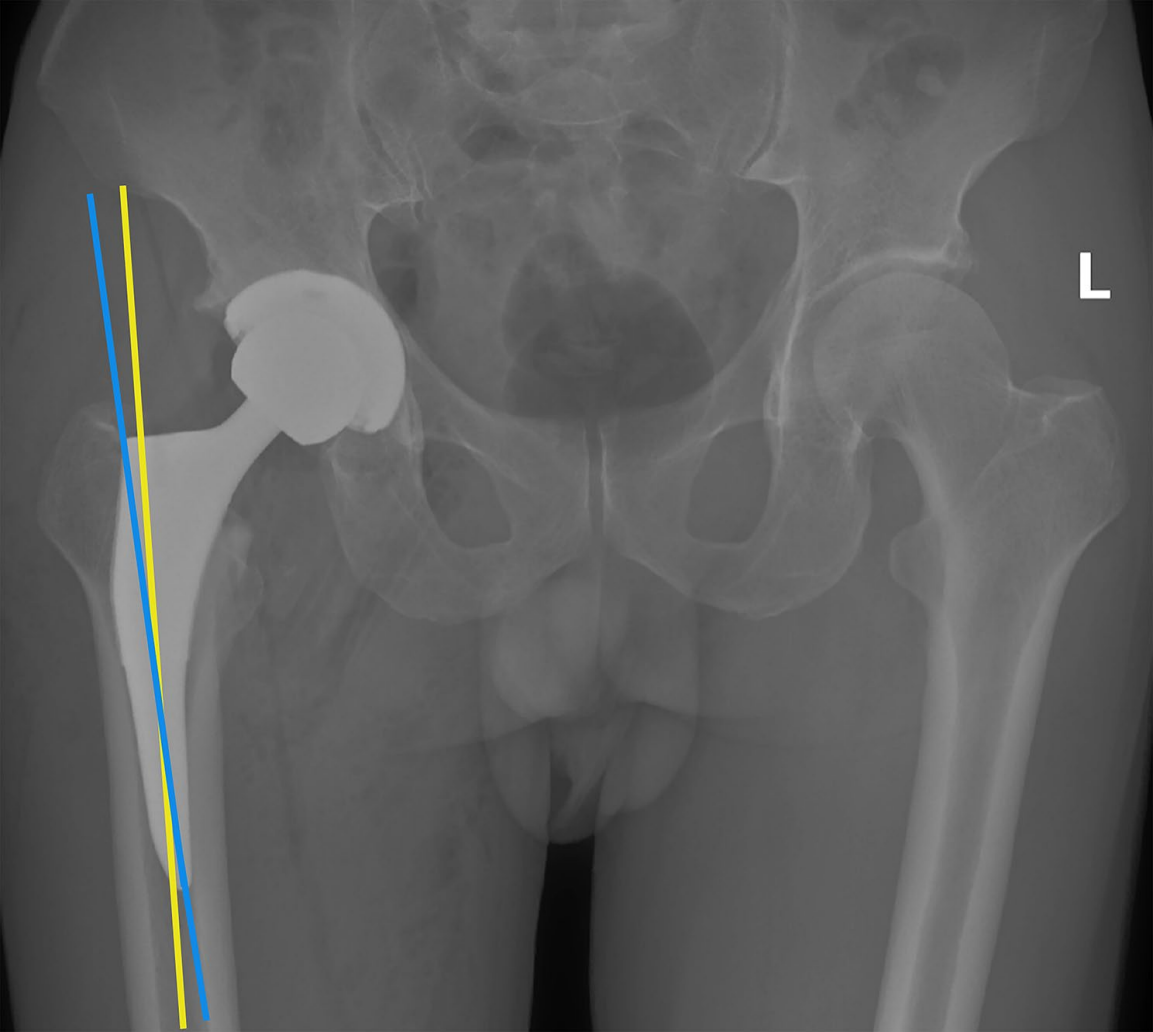
**The angle between the femoral and stem.** The blue line represents the mechanical shaft of the femoral stem and the yellow line represents the mechanical shaft of the femoral

**Fig. 5:**
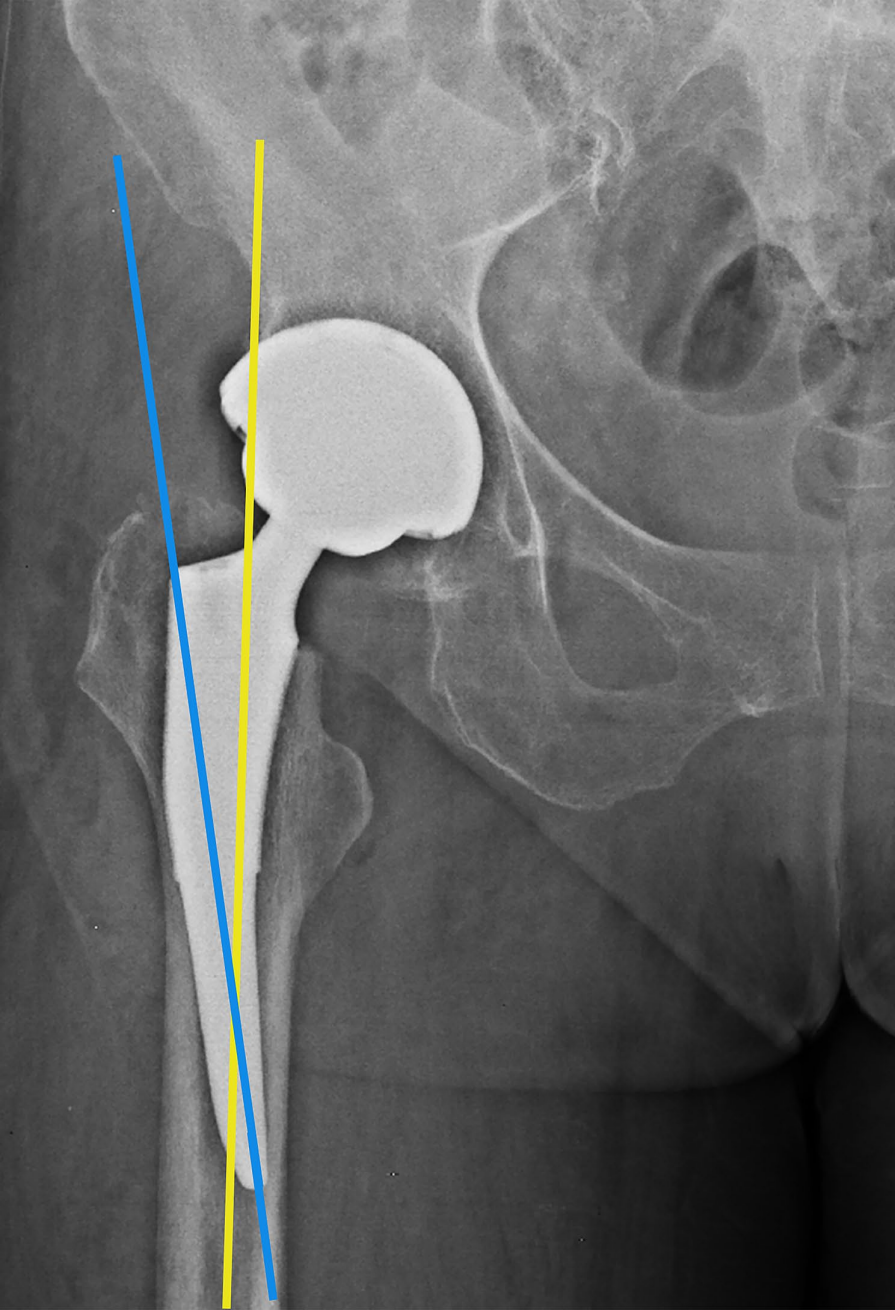
**Severe femoral stem malalignment.** Excessive angle between the femoral and the stem, resulting in valgus of the stem

Postoperatively, the angle between the femoral and the stem was measured on the DR film of the anteroposterior hip joint (Fig. 4), and that greater than 3°was considered as stem malalignment [26] (Fig. 5).

### Body mass index

BMI was primary obtained by calculating the general admission data of patients.

### WOMAC

WOMAC score obtained according to the last follow-up [27] (Scores were counted as none = 1, mild = 2, moderate = 3, severe = 4, and extreme = 5).

### Statistical analysis

Data analysis was performed on SPSS (version 23.0, IBM Corporation, New York, NY, USA) statistical software. Descriptive analysis was conducted on each variable, with the means were expressed as ±s. Univariate significance for comparisons between the stem malalignment group and neutral position groups was obtained by performing independent samples t-test for continuous variables and chi-square test for categorical variables. The risk factors for femoral stem malalignmet were then expressed depending on the modified Poisson regression (Outcome events are more common (> 10%)) (considering that the postoperative follow-up score and preoperative diagnosis were not significantly correlated with the placement angle of the femoral prosthesis, so they were not entered into the equation) with the odds ratio (OR) and 95% confidence interval (CI). Differences were considered statistically significant at P<0.05.

## Results

### Analysis of the number of participants

The retrospective analysis was carried out on a total of 160 patients with initial total hip replacement, among whom 113 patients were finally enrolled based on the inclusion criteria, with 47 patients in the femoral stem malalignment group and 66 patients in the neutral position group.

### Differences in clinical and imaging parameters

The cases included 52 males (46.0%) and 61 females (54.0%), aged 23–84 years, with an average of (57 ± 14) years. Preoperative diagnoses were: Osteoarthritis (73.5%), Avascular necrosis (21.2%), Femoral neck fracture (4.4%) and Rheumatoid arthritis (0.9%). The patients could be divided into femoral stem malalignment group and neutral position group according to imaging measurements. With the comparison in the clinical and imaging parameters, significant differences was demonstrated in FAR ratio was used during surgery between the two groups (Table 1). The results indicated the FAR ratio<1 in 20 of 47 study subjects (42.6%) in the femoral stem malalignment group and <1 in 42 of 66 study subjects (63.6%) in the neutral position group, with a statistically significant difference between them (χ^2^ = 4.93, P<0.05). No significant difference was found in other parameters such as gender, age, BMI, preoperative diagnoses, Dorr classification, pelvic morphology ratio>3 and WOMAC score at the last follow-up between the femoral stem malalignment group and the neutral position group (P>0.05).

**Table 1:** The relationship between different influencing factors and the malalignment of the femoral stem

Factors	Variable declaration	Femoral stem malalignment (n = 47)	Neutral position (n = 66)	χ^2^ or t value	P value
Gender	Male	22 (46.80)	30 (45.50)	0.02	0.88
	Female	25 (53.20)	36 (54.50)		
Age (years)		56 ± 14	58 ± 14	0.96	0.34
BMI		23.88 ± 2.99	25.17 ± 3.78	1.95	0.05
Pelvic morphology	<3	13 (27.70)	15 (22.70)	0.36	0.55
	>3	34 (72.30)	51 (77.30)		
FAR ratio	<1	20 (42.60)	42 (63.60)	4.93	0.03*
	>1	27 (57.40)	24 (36.40)		
Preoperative diagnoses	Osteoarthritis	35 (74.50)	48 (72.80)	4.90	0.17
	Avascular necrosis	9 (19.10)	15 (22.70)		
	Femoral neck fracture	3 (6.40)	2 (3.00)		
	Rheumatoid arthritis	0	1 (1.50)		
Dorr classification	Type A	6 (12.80)	8 (12.10)	0.01	0.92
	Type B	41 (87.20)	58 (87.90)		
Last follow-up WOMAC		26.11 ± 3.20	26.41 ± 4.33	0.41	0.69

*Significant difference

### Risk factors affecting femoral stem malalignment

Considering no significant correlation between WOMAC at the last follow-up, preoperative diagnoses and femoral stem placement angle, it was temporally not added into the equation for the time being. The remaining factors were explored according to regression analysis to further investigate the risk factors affecting femoral stem malalignment. The regression equation showed that BMI (OR = 1.01, 95% CI: 1.00-1.03, P<0.05), FAR ratio<1 (OR = 1.15, 95% CI: 1.03–1.28, P < 0.05) as significant risk factor for femoral stem malalignment in patients undergoing DAA-THA, (Table 2).

**Table 2:** **Risk factors for femoral stem malalignment.** Univariable and multivariable analysis were performed to determine the correlation between each factor and femoral stem malalignment

Varible	Univariable analysis		Multivariable analysis	
	OR (95% CI)	P-value	OR (95% CI)	P-value
Gender(male relative to female)	0.99(0.88–1.11)	0.89	0.90(0.86–1.12)	0.76
Age (change per year increase)	1.00(0.99–1.01)	0.35	1.00(0.99–1.01)	0.39
BMI(change per unit indext increase)	1.01(1.01–1.03)	0.01*	1.01(1.00-1.03)	0.04*
Pelvic morphology(>3 relative to <3)	1.04(0.91–1.20)	0.56	1.08(0.94–1.25)	0.29
FAR ratio(<1 relative to >1)	1.14(1.02–1.28)	0.03*	1.15(1.03–1.28)	0.02*
Dorr classification(A relative to B)	0.99(0.83–1.18)	0.92	1.04(0.81–1.34)	0.76

*Significant difference

### Intraoperative use of curved rasp with stem malalignment and complications

The 113 THA cases were divided into Group A (with) and Group B (without) according to whether the use of curved rasp during surgery. When the baseline characteristics of population were not significant(gender, age, BMI), the clinical and imaging parameters of the two groups were compared, and it was found that the use of curved rasp and the placement angle of prosthesis had statistical significance (P<0.05) (Table 3).

**Table 3:** The relationship between different factors and the use of curved rasp

Factors	Variable declaration	Group A (n = 37)	Group B (n = 76)	χ^2^ or t value	P value
Gender	Male	17 (45.90)	35 (46.10)	0	0.99
	Female	20 (54.10)	41 (53.90)		
Age		55 ± 15	58 ± 14	1.03	0.31
BMI		25.49 ± 3.42	24.22 ± 3.51	1.83	0.07
Femoral stem implantation angle	Femoral stem inversion	10 (27.00)	37 (48.70)	4.81	0.03*
	Neutral position	27 (73.00)	39 (51.30)		

*Significant difference

**Fig. 6:**
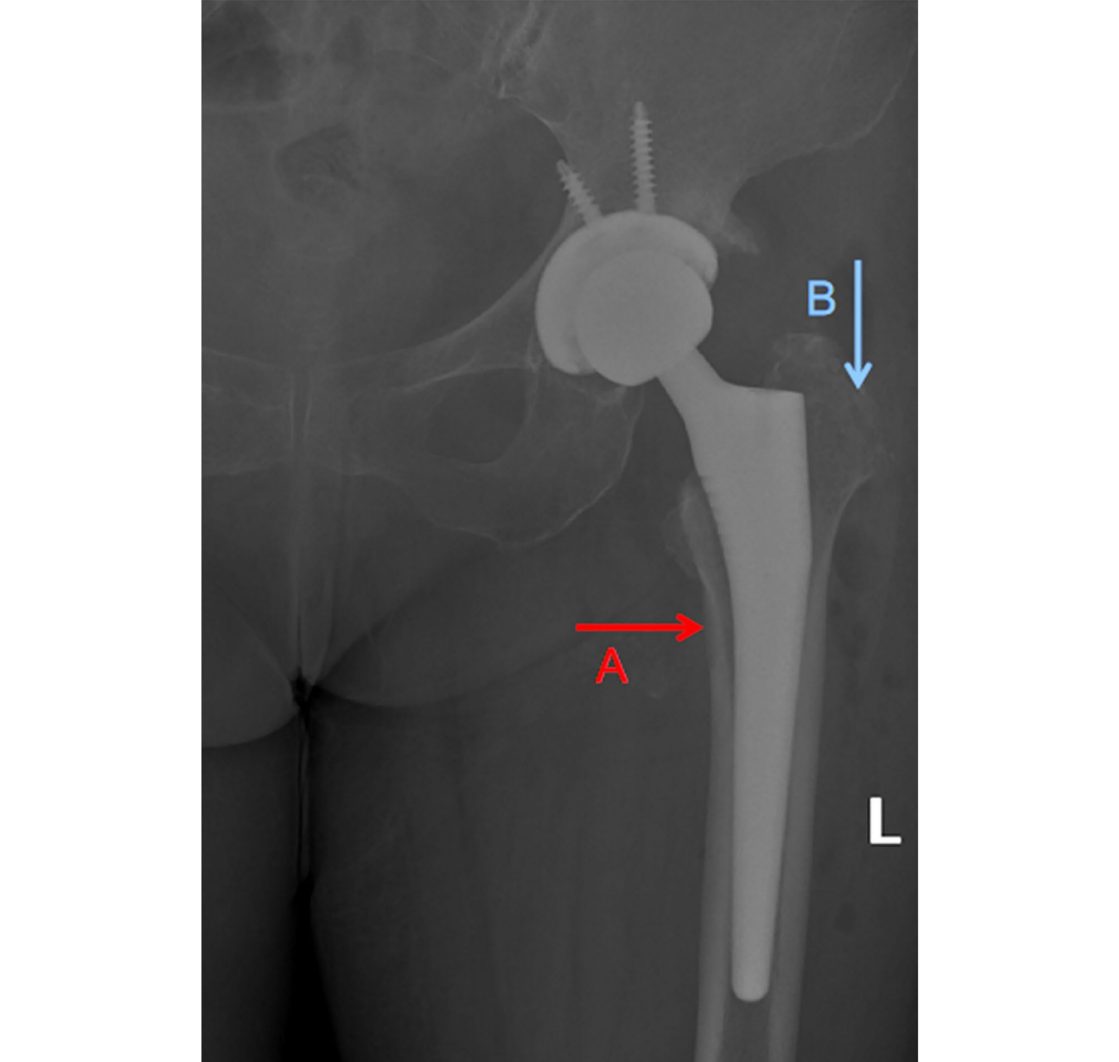
**Intra-operative complications.A** is the femoral side perforation, **B** is excessive removal of lateral cancellous bone

The odds of femoral stem malalignment were 27.0% in group A, while the likelihood of femoral stem inversion malalignment was significantly increased to 48.7% in group B (P<0.05). Furthermore, the relationship between the intraoperative use of curved rasp and the prosthesis placement angle was investigated. The prosthesis placement angle was categorized into neutral (<3°), mild (≥ 3° and <4°), moderate (≥ 4° and <5°), severe (≥ 5°), the difference was statistically significant revealed by Fisher’s exact test (P<0.05) (Table 4). The intraoperative poor preparation of the femoral side will affect the angle of femoral prosthesis placement and even the occurrence of fracture and perforation (Fig. 6), so the relationship between the occurrence of adverse outcomes and the use of curved rasp was also investigated in this study, but no statistically significant difference was found (P>0.05) (Table 4).

**Table 4:** Relationship between femoral stem implantation angle, intraoperative complications and the application of curved rasp

Factors	Variable declaration	Group A (n = 37)		Group B (n = 76)	P value
Femoral stem implantation angle	Neutral (<3°)	28 (75.70)	39 (51.30)		0.02*
	Mild (≥3°and<4°)	0	7 (9.20)		
	Moderate(≥4°and<5°)	8 (21.60)	19 (25.00)		
	Severe (≥5°)	1 (2.70)	11 (14.50)		
Intraoperative complications	Femur fracture or perforation	37 (100.00)	74 (97.40)		1.00
	None	0	2(2.60)		

*Significant difference

## Discussion

Postoperative pain, aseptic loosening, subsidence, and even fractures resulting from the angle of implant placement after orthopedic surgery have always been the focus for orthopedic surgeons. More studies are focused on the issues of acetabular impingement, bone impingement, prosthesis loosening and subsidence after THA [1–3, 28, 29], while surgical instrumentation and femoral stem malalignment were rarely mentioned. In this retrospective study with a relatively limited number of patients, the key finding was the more satisfactory radiographic outcomes with the use of a curved rasp in patients with a Tri-lock (BPS) stem for initial THA in DAA.

Concerns have been raised to relatively high rate of coronal malalignment of short-stemmed prostheses compared to traditional standard-length femoral prostheses [26]. Indeed, the lack of proper distal extension into the diaphysis in short stems impedes the surgeon to correctly guide them during implantation. Varus-valgus malalignment is a critical factor in the use of cementless stems to avoid complications [30, 31]. Vresilovic et al. demonstrated that varus alignment correlated with loosening of cementless stems [30], and Gill et al. demonstrated that varus alignment caused periprosthetic femoral insufficiency fractures [31]. However, as a systematic evaluation performed by Lidder et al. [32] revealed no significant difference was reported in survival and clinical outcomes with respect to femoral stem malalignment compared to neutral position. Several studies maintained a deviation of 5°from neutral as the definition of varus and valgus dislocation malalignment [33]. In the present study, the cases of inversion malalignment occupied a quite high proportion, as we used a threshold of 3° [16], with the average varus or valgus angle measured as 1.78°. While if 5°was selected as the threshold for varus or valgus dislocation, only 12 patients (10.6%) were outliers. During the DAA operation, the exposure and lifting of the proximal femur undoubtedly lies the most challenging stage of the operation. The insufficient preparation on the femoral side may result in deviation of the prosthesis placement angle with a high probability, even perforation or fracture of the femoral medullary cavity.

During supine manipulation, the emergence of any inadequate lateral release of the femur or substandard preparation of the femoral medullary canal can induce the malalignment of femoral component, particularly the stem valgus or varus [34]. Influenced by insufficient elevation of the proximal femur, the inconsistent direction of reaming to the direction of the medullary canal, the following manipulation will result in varus implantation of the femoral stem [35], especially in the case of high BMI and low FAR [5]. And our designed curved rasp can not only achieve to run direction of the femoral medullary cavity be found when the proximal femur is not fully lifted, but also repeatedly remove the cancellous bone in the posterior femoral opening to ensure the accuracy of the femoral prosthesis implantation, avoiding the incidence of intraoperative medullary perforation or fracture. Despite the only 2 cases (2.6%) of adverse outcomes in the group without using curved rasp, compared with the group using one. There is no significant statistical difference, but it still has a certain clinical guiding significance.

According to Batailler et al. [36], we noted was less varus malalignment when a more aggressive and less prominent lateral shoulder puller was used via DAA. In this study, the probability of femoral stem malalignment was significantly reduced in used the curved rasp group compared unused group, the statistical outcomes revealed the probability of malalignment of the femoral stem of 27.0% in patients who used curved rasp during surgery, increased to 48.7% in femoral stem malalignment(P<0.05), which also confirmed Batailler’s view. In this study, we further investigated the association of intraoperative use of curved rasp with prosthesis placement angle, with the prosthesis placement angle divided into neutral (<3°), mild (≥ 3°and<4°), moderate (≥ 4°and<5°), severe (≥ 5°). According to the results of Fisher’s exact test, the angle of prosthesis placement was indicated better in group A than in group B, especially in mild (0%) and severe (2.7%) malalignment, with the statistically significant difference (P<0.05).

The negative impact of BMI on THA prognosis has been widely documented in relation to infection, instability, increased operative time, and a lot of other complications [25, 37, 38]. Obese patients are more prone to serious complications after surgery, so the hip replacement surgery is not recommended in patients with a BMI greater than 40 kg/m2, according to American Society of Knee and Hip Physicians [39]. Little evidence is available regarding the effect of BMI on the incidence of poor prosthesis positioning. However, it was indicated that the incidence of femoral stem malalignment was increased in line with the patient BMI, which was further confirmed by regression analysis. This provides orthopaedic surgeons reason for renewed vigilance when selecting suitable patients for hip replacement.

The shape of the pelvis was proposed in the work of Rubin et al. [8], that is, when the ratio of the line connecting the widest part of the ilium and the most distal part of the ischium, is>3:1, the iliac wing is more likely to hinder the implantation of the stem. There still lacks sufficient clinical evidence and research to validate this view, which may be related to the differences of different ethnic groups in experience of the surgeon or the physiological and anatomical. In the present study, no significant difference was found in the ratio of pelvic morphology between the femoral stem malalignment group and the neutral position group (P>0.05), which is supposed to be further analyzed in the follow-up study.

To date, multiple morphological bone features have been demonstrated to affect stem alignment. Wang et al. [6] have concluded the impacts of femoral valgus, finding that GT protrusion beyond the midline of the femur will significantly increase the risk of coronal dislocation. Sheridan G et al. [5] validated the desirability of the application of ratiometric measurements instead of absolute measurements as it bypass the requirement to incorporate radiographic magnification into the measurements taken. It was also shown that FAR ratio<1 resulted in higher prosthesis misalignment. In the present study, the probability of stem inversion was predicted by the FAR ratio (OR = 1.15, 95% CI: 1.03–1.28, P<0.05), it just confirms that view. That is to say, FAR ratio<1 is 1.15 times more likely to result in varus or valgus stem malalignment.

### Limitations of the study

This study is inevitable to certain limitations. First, this is a retrospective study with a relatively limited number of patients undergoing DAA primary THA with a curved rasp utilized in Tri-lock (BPS) stem. However, the number of patients in each group provided sufficient robustness for the comparison in radiological outcomes rather than functional assessments and complications. The preoperative functional scores and variable last follow-up time varied among patients, and the WOMAC functional score was weak for us to draw meaningful conclusions, that any type of prosthesis indicated good results within 5 years. Furthermore, our objective was to explore the early results, especially the radiological outcomes associated with the use of DAA in primary THA using contusion. Second, when applying the medical image archiving and communication system or Mimics 20.0 in observing the frontal and lateral X-rays of the hip joint before and after surgery, taking the average of the two groups for abnormal data may cause overall data bias considering the data deviation due to personal subjective reasons. These potential biases may limit the generalizability of the findings to other populations. Another limitation of this study is that all procedures were carried out at one center by an orthopaedic surgeon and his medical team experienced with the DAA learning curve, which means that the findings may reflect the experience of only one orthopaedic surgeon. Therefore, further multicenter studies are required to obtain more consistent results.

## Conclusions

In DAA-THA, BMI and FAR ratio<1 act as the independent risk factors for femoral stem malalignment. The intraoperative use of a curved rasp for lateral femoral preparation could significantly reduce the incidence of malalignment of the femoral stem.

## Electronic supplementary material

Below is the link to the electronic supplementary material.

Supplementary Material 1

## Data Availability

The datasets used and/or analyzed during the current study are available from the corresponding author on reasonable request; please contact the corresponding author, Dr. Feng. Administrative permission was received from Department of Arthrosis Surgery, Fuzhou Second Hospital, Fuzhou, China (No. 47, Shangteng Road, Cangshan District, Fuzhou, China) to access the medical records.

## Abbreviations

DAA-THA: Direct anterior approach total hip arthroplasty
DAA: Direct anterior approach
THA: Total hip arthroplasty
FAR: Femoral access ratio
BMI: Body mass index
WOMAC: Western Ontario McMaster Universities (WOMAC) Osteoarthritis Index
OR: Odds ratio
CI: Confidence interval
